# Polydopamine-Based Molecular Imprinting Polymer Electrochemical Sensor for Neopterin Detection

**DOI:** 10.3390/bioengineering13040416

**Published:** 2026-04-02

**Authors:** Elena Dilonardo

**Affiliations:** Institute of Nanotechnology, CNR-NANOTEC, Via G. Amendola, 122, 70125 Bari, Italy; elena.dilonardo@cnr.it

**Keywords:** biomarker, neopterin, molecularly imprinted polymer, polydopamine, electrochemical biosensor

## Abstract

Neopterin, a low-molecular-weight pteridine, is a biomarker of pro-inflammatory immune activity. Its levels rise in viral infections, transplant rejection, autoimmune, cardiovascular, and neurodegenerative diseases, and cancer. In healthy human serum, neopterin concentration values are up to 10 nM. Detection is challenging due to its low concentration and limited solubility. In this work, a sensitive and selective electrochemical sensor for neopterin was developed using polydopamine molecularly imprinted polymers on a glassy carbon electrode. The polymer films were electro-polymerized directly on the electrode, varying the ratio of polymer to neopterin, while non-imprinted films were prepared without the template for comparison. Rebinding and template removal were monitored by cyclic voltammetry using ferricyanide as a redox probe. All imprinted films exhibited a concentration-dependent response from 1.2 nM to 1.2 mM, with a rapid increase at low concentrations up to 120 nM and a slower approach to a plateau at higher concentrations. The highest response was observed in films with the greatest neopterin content, consistent with increased binding site availability.

## 1. Introduction

Biomarkers are indicators that measure and evaluate in biological fluids (e.g., blood serum and urine) normal biological processes, pathogenic processes, or pharmacologic responses to a therapeutic intervention [1]. Indeed, nowadays, biomarkers are more often used to predict risk, to screen, to monitor, and to diagnose [2,3,4]. Recently, biomarkers have become indispensable tools in biomedicine thanks to their ability to inform about an individual’s health status quantitatively and specifically [5,6,7]. Therefore, selective and sensitive biomarker determination is critical. In this context, neopterin, also known as 1′,2′,3′-D-erythro-trihydroxypropylpterin, is a small-weight molecule, well-recognized, pro-inflammatory immune response biomarker [8]. It is an unconjugated pteridine found as a metabolic product in living cells [9,10]. The increase in the neopterin concentration in the human body is associated with increased production of reactive oxygen species [11], related to the activation of the cellular immune response in the progression of various diseases such as virus infections, such as HIV-1 and COVID-19, transplant rejection, autoimmune disorders, cardiovascular and neurodegenerative diseases, malignant tumours, and many other pathologies [12,13]. Neopterin’s high sensitivity to light limits its stability and solubility in biological fluids; therefore, the low solubility and low concentration complicate its analysis [10,14,15]. In serum, the neopterin concentration values are up to 10 nM in healthy individuals, while higher than 10 nM can be considered elevated [15,16]. Up to now, various analytical techniques have been used to detect neopterin in blood, mainly high-pressure liquid chromatography (HPLC) [17], radioimmunoassays (RIA) [18], fluoroimmunoassay [19], and enzyme-linked immunosorbent assay (ELISA) [20]. However, all these methods are time-consuming, tiring, and expensive; moreover, they require complex technical equipment and trained staff, and their validity and quality can be affected by adverse factors [21,22]. Therefore, alternative methods are urgently desirable. Recently, among various fluorescent sensors with quick response, high sensitivity, and simple operation, lanthanide complexes show distinctive luminescence properties. J. Zhao et al. [22] have reported the use of Zn(II)-Eu(III) nanocluster for quantitative luminescence detection of neopterin concentrations in fetal calf serum and urine with limit of detection (LOD) values of 4.3 nM and 2.5 nM, respectively. Some limitations associated with the employment of the lanthanide complexes and nanoparticles include the low selectivity [23] and the difficulty of being directly excited [24]. The present successes in nanotechnology and instrument development have led to the recognition of biosensing systems based on surface-enhanced Raman spectroscopy (SERS) with a higher sensitivity towards bioanalytes. In the literature, SERS has been used to detect neopterin in cerebrospinal fluid with an LOD of 1.6 nM, as reported in Ref. [21], and in blood with an LOD of 1.4 nM in Ref. [25]. Up to now, the key technical problems to be solved in the application of SERS for biosensing are the production of a uniform substrate and reproducible assays [26,27].

Recently, C. Hong et al. [28] have developed an on−off ratiometric fluorescence Lanthanide Metal−Organic Framework (Ln-MOF) material based on Europium (III) for neopterin detection with an LOD of 15.11 nM; in this case, although the promising results, the commercialization of Eu(III)-MOFs-based neopterin senso is limited by the challenges of scaling up synthesis to industrial levels, including long synthesis times, high costs, the use of toxic solvents, and difficulties in achieving consistent quality control and purification [29]. Therefore, nowadays, to respond to the more and more compelling necessity for fast and accurate detection of cancer biomarkers, the precise development of a device suitable for this purpose plays a crucial role in the effectiveness and efficiency of the device itself in terms of selectivity, cost, LOD, and analysis time. In this context, molecular imprinted polymer (MIP)-based biosensors appear as promising tools for point-of-care testing due to low cost, ease of miniaturization, and the possibility of integration with multi-array tools, also exploiting different detection techniques [13,30,31,32]. MIPs are usually made up of functional monomers, which are polymerized in the presence of the target (template) molecules in an appropriate solvent. Firstly, a pre-polymerization complex is formed by the target and the functional monomer molecules through covalent or non-covalent interactions, followed by the polymerization process [33]. The subsequent removal of the template molecules from the polymer matrix leaves behind empty binding sites, which possess high selectivity towards the template [34]. The molecular memory imprinted on the polymer, consisting of the correct shape and orientation of functional groups, permits the selective binding of the target, allowing a selective recognition of the imprint species [8]. In this context, MIP-based electrochemical biosensors appear as promising tools for point-of-care testing due to low cost, ease of miniaturization, and possibility of integration with multi-array tools [13]. P.S. Sharma et al. [12] have used a conducting polymer mix of bis–bithiophene derivatised with cytosine and bithiophene derivatised with boronic acid on a platinum electrode as an MIP-based potentiometric chemosensor to detect neopterin with an LOD of 22 μM. However, the detection potential of this method was high, and its sensitivity was limited; therefore, looking for excellent polymeric monomers is crucial to improve the sensitivity and selectivity towards the neopterin detection. Recently, A. Zengin et al. [35] have reported the use of magnetic silica-supported MIP (MIP-MS) sensors, offering one of the lowest LODs (1.2 nM) for neopterin using a MIP approach, with good linear response range and repeatability; although, this system suffers from complex synthesis, potential template leakage, limited aqueous compatibility, and lacks real-time detection capability.

Table 1 summarizes previously reported neopterin detection types, including their recognition method, concentration detected range, LOD, and main advantages/limitations.

In the present work, dopamine (3,4-dihydroxy phenethylamine) was used as a functional monomer, a non-conducting catecholamine. Thanks to the multifunctionality, high stability, simple but finely tunable film deposition on various materials (e.g., metals, ceramics, plastics) via electro-polymerization [36,37], a facile electrochemical procedure was proposed to prepare a polydopamine molecular imprinted polymers (PDA-MIPs)-modified glassy carbon electrode as electrochemical sensor to detect neopterin; indeed, firstly, the electrochemical polymerization of dopamine in presence of the target neopterin molecule was involved, and then its removal from the prepared MIP film. By comparison, the non-imprinted polymer (PDA-NIP) films were synthesized by exploiting the same procedure but in the absence of the template. MIP synthesis, template removal, and neopterin rebinding have been monitored by cyclic voltammetry (CV) using ferricyanide as a redox marker. Under optimized conditions, this molecularly imprinted electrochemical sensor presented suitable sensitivity to detect neopterin with an LOD lower than 1.0 nM.

Finally, the aim of this study was to demonstrate the feasibility of an electropolymerized PDA-based MIP as an electrochemical sensor for neopterin detection and to evaluate its analytical performance in terms of sensitivity and response behaviour.

## 2. Materials and Methods

### 2.1. Reagents

Dopamine (DA) hydrochloride (p.a. purity ≥ 97.5%), D-neopterin (p.a. purity ≥ 97.5%), tris(hydroxymethyl)aminomethane (TRIS) (ultra pure ≥ 99.8%), potassium ferrocyanide trihydrate, (K_4_Fe(CN)_6_∙3H_2_O, ≥98.5%), potassium ferricyanide (K_3_[Fe(CN)_6_, 99%), potassium chloride (KCl, ≥99.0%), hydrochloride (HCl, 37%w) were purchased from Sigma-Aldrich (St. Louis, MO, USA) and used as received without further purification. All solutions are prepared with ultrapure water (Millipore Milli-Q, Bedford, MA, USA, 18.2 MX cm^−1^).

TRIS–HCl buffer (denoted as TRIS buffer) pH 7.2 was prepared by dissolving TRIS base in water and adjusting the pH by adding HCl.

Ferro-ferricyanide solution for CV was prepared by dissolving K_4_[Fe(CN)_6_] and K_3_[Fe(CN)_6_] in supporting electrolyte solution (0.1 M KCl).

### 2.2. Electrochemical Deposition

Before the electro-polymerization procedure, glassy carbon (GC) electrodes, on which the electro-depositions would be performed, were firstly rubbed down on sandpaper with different granularity and then with alumina powders, successively ultrasonically cleaned with Milli-Q water and alcohol, and dried at room temperature.

Electrochemical experiments for electro-polymerization were carried out with a CHI 660D Potentiostat (CH Instruments, Bee Cave, TX, USA) controlled by a computer. A one-compartment three-electrode cell was used, consisting of the GC electrode (3 mm diameter) as working, a platinum wire as counter electrode, and Ag/AgCl (3 M KCl) as reference electrode.

Cyclic voltammetry (CV) was used to electropolymerize both DA alone, defined as non-molecularly imprinted polymer (NIP-PDA), and DA in the presence of the target neopterin molecule, defined as molecularly imprinted polymer (MIP-PDA). at different concentration ratios, onto the previously cleaned GC electrodes.

**Figure 1:**
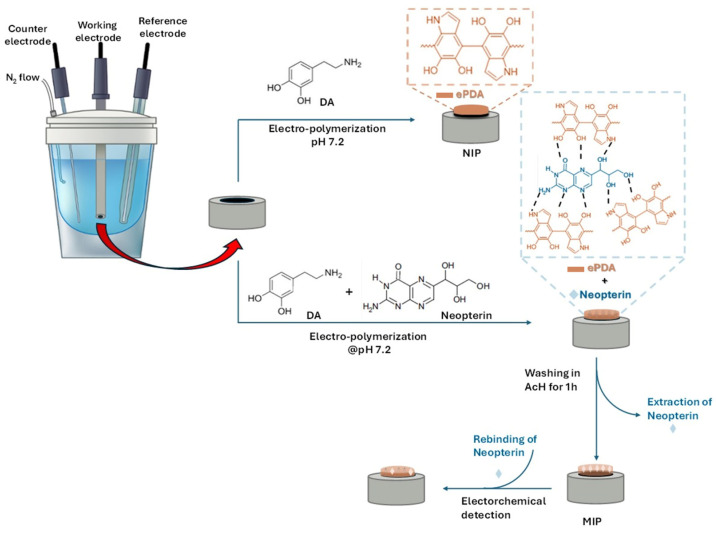
Scheme of the electrochemical preparation of NIP-PDA and MIP-PDA for the electrochemical detection of neopterin.

In Figure 1, a scheme of the electrochemical procedure to obtain NIP-PDA and MIP-PDA is reported.

**Figure 2:**
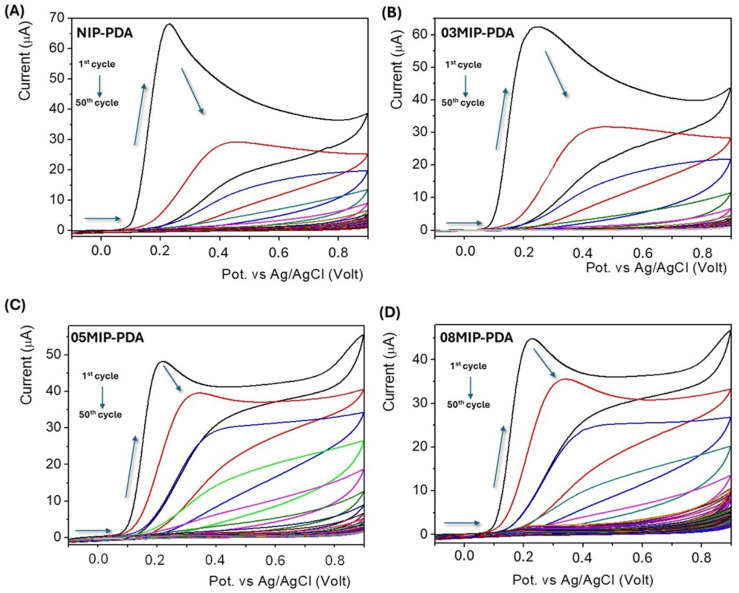
Cyclic voltammograms for the electro-polymerization on GC electrode in TRIS buffer (pH 7.2) containing (**A**) only 1 mg mL^−1^ DA (NIP-PDA), (**B**) 1 mg mL^−1^ DA and 0.3 mg mL^−1^ neopterin (03MIP-PDA), (**C**) 1 mg mL^−1^ DA and 0.5 mg mL^−1^ neopterin (05MIP-PDA), (**D**) 1 mg mL^−1^ DA and 0.8 mg mL^−1^ neopterin (08MIP-PDA), respectively; scan rate: 20 mV s^−1^; number of scans: 50. Different colors are used only to distinguish the individual CV cycles during electrodeposition, while the arrows indicate the scan direction.

Specifically, NIP-PDA was obtained by electro-polymerization on the GC working electrode in an aqueous solution of 1 mg ml^−1^ DA, buffered at pH 7.2, and under nitrogen flux to avoid possible contribution of alkaline oxidative auto-polymerization of DA. Electrochemical polymerization was performed at −0.1 to 0.9 V in the fresh DA-Tris solution (scan rate: 20 mV s^−1^; number of scans: 50). In Figure 2A, the cyclic voltammograms of electro-polymerization of DA to obtain NIP.

MIP-PDA was obtained following the same procedure used for the preparation of NIP-PDA, with the addition of the target neopterin molecule at the following three different concentrations, 0.3, 0.5, and 0.8 mg mL^−1^. These correspond to neopterin/DA molar ratios of 0.3, 0.5, and 0.8, respectively, and the resulting materials were labelled as 03MIP-PDA, 05MIP-PDA, and 08MIP-PDA. The template concentrations selected for the preparation of the MIP-PDA films were chosen to cover a practical and representative range for systematic investigation of the effect of template amount on sensor performance. Exploring multiple template concentrations is a common strategy in the optimization of molecularly imprinted polymer sensors to obtain a clear trend in sensitivity and binding capacity, and to avoid excessive template that could lead to polymer inhomogeneity or hindered analyte access to binding sites [38,39,40]. Similar approaches, where the monomer/template ratio and template concentration are varied to evaluate their impact on the formation of binding sites and analytical response, have been used in recent electrochemical MIP sensor studies [38,39,40].

Figure 2B–D shows the electrochemical polymerized curves of DA on the GC electrode by CV in the presence of 0.3 mg mL^−1^ neopterin (03MIP-PDA), 0.5 mg mL^−1^ neopterin (05MIP-PDA), and 0.8 mg mL^−1^ neopterin (08MIP-PDA), respectively.

In all reported voltammograms in Figure 2, in the first CV cycle, the first anodic peak at 0.25 V (the corresponding cathodic peak has not been acquired since the scan range was restricted to reduce the deposition time, permitting to obtain a stable and homogenous electrodeposited film) indicates the initial presence of non-oxidated DA; increasing the number of the scans, the peak current decreases, indicating the creation of an insulating electrodeposited polydopamine (ePDA), electrochemically inactive that prevents a further electrochemical oxidation of DA at electrode-solution interphase [41].

In the presence of neopterin, DA monomers electro-polymerize around the template molecules, resulting in the formation of imprinted sites possessing a specific affinity for the target molecule (Figure 1).

After the electro-depositions, in all cases, the electrodes were washed in the following three sequential steps: deionized water, acetic acid (5%v) to remove the template molecules and other adsorbates, and finally again deionized water before drying with a stream of nitrogen. Each wash lasted for 1 h under gentle stirring.

### 2.3. Characterization Techniques

The chemical surface of modified electrodes was analyzed by X-ray Photoelectron Spectroscopy (XPS) using an Axis ULTRA DLD Spectrometer (Kratos Analytical, Manchester, UK) with a monochromatic Al Kα source operating at 225 W (15 kV, 15 mA). For each sample, a wide scan spectrum was acquired in the binding energy range of 0–1200 eV with a pass energy of 160 eV and a 1 eV step, while high-resolution regions were acquired with a pass energy of 20 eV and a 0.1 eV step. In both cases, the area of analysis is about 700 × 300 μm^2^. The base pressure in the instrument was 1 × 10^−9^ mbar. Data analysis was performed by CasaXPS software (http://www.casaxps.com, accessed on 15 January 2026). Charge correction of the spectra was performed considering the alkyl-type carbon (C-C, C-H) component of the C 1 s spectrum as an internal reference (Binding Energy, B.E.  =  285.0 eV). For each sample, XPS analysis was repeated at five different points to evaluate the process reproducibility.

The presence of the insulating ePDA, which uniformly covered the modified electrodes in NIP-PDA and irregularly in MIP-PDA after template removal, was evaluated by cyclic voltammetry (CV), using K_4_[Fe(CN)_6_]/K_3_[Fe(CN)_6_] as red-ox probe in TRIS buffer pH 7.2.

To evaluate the binding properties of the MIP sensors, the modified electrodes were incubated for 1 h in TRIS buffer (pH 7.2) containing neopterin at progressively increasing concentrations, ranging from 1.2 nM to 1.2 mM. At each analytical step, a higher concentration of neopterin was used to assess the sensor’s response. After incubation, the electrodes were rinsed with TRIS buffer to remove any non-specifically adsorbed neopterin from the sensor surface. The MIP electrodes were then electrochemically characterized by cyclic voltammetry (CV) using a [Fe(CN)_6_]^3−^/^4−^ redox probe at a scan rate of 50 mV s^−1^ in TRIS buffer solution (pH 7.2).

## 3. Results

**Figure 3:**
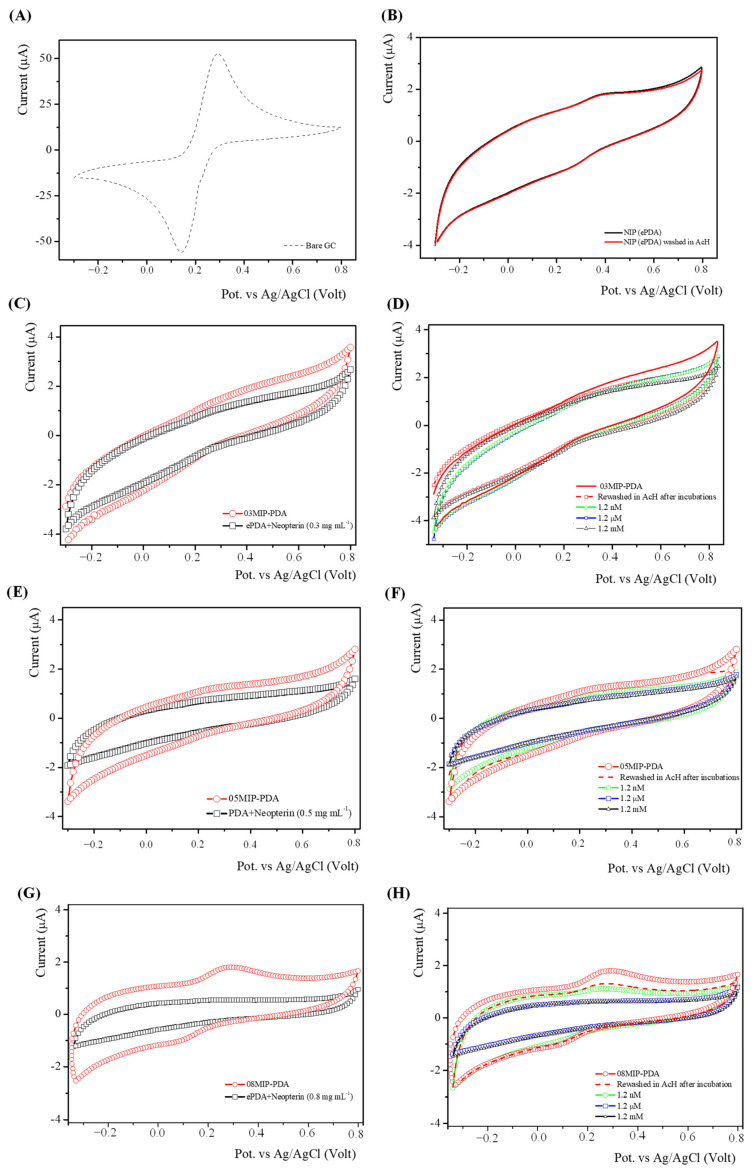
Cyclic voltammograms of the [Fe(CN)_6_]^3−^/^4−^ redox couple obtained at modified GC electrodes: (**A**) Bare GC electrode used as the reference; (**B**) NIP; (**C**) 03MIP-PDA before and after template removal (0.3 mg mL^−1^ neopterin); (**D**) 03MIP-PDA recorded before and after incubation in neopterin solutions with increasing concentrations; (**E**) 05MIP-PDA before and after template removal (0.5 mg mL^−1^ neopterin); (**F**) 05MIP-PDA recorded before and after incubation in neopterin solutions with increasing concentrations; (**G**) 08MIP-PDA before and after template removal (0.8 mg mL^−1^ neopterin); (**H**) 08MIP-PDA recorded before and after incubation in neopterin solutions with increasing concentrations.

The redox probe [Fe(CN)_6_]^3−^/^4−^ was employed to investigate the electrochemical properties of the modified electrodes. Figure 3 shows the cyclic voltammograms recorded in the probe solution for all prepared electrodes, compared with the bare GC electrode. In all cases, for NIP and e-PDA electrodes prepared in the presence of neopterin before template removal, weaker current peaks were observed, indicating that the e-PDA layer effectively coated the GC electrode surface.

Specifically, for MIPs, current values recorded before neopterin template removal were the lowest, whereas after template removal, a significant increase in current was observed. The voltammogram of the unmodified bare GC electrode exhibits two well-defined peaks corresponding to the [Fe(CN)_6_]^3−^/^4−^ redox process, characteristic of a diffusion-controlled process. After electro-polymerization of dopamine in the presence of neopterin (ePDA + Neopterin), no redox peaks were observed. Following neopterin removal in acetic acid, the voltammetric response of [Fe(CN)_6_]^3−^/^4−^ was partially restored. Upon incubation of the MIP sensor with neopterin at increasing concentrations, the sensor response decreased. After the final acetic acid washing, the current increased slightly but did not reach the initial value.

To evaluate the response of the MIP-modified electrodes to different neopterin concentrations, the anodic peak current at 0.31 V was monitored. The sensor response was expressed as the normalized current variation, calculated as follows:

(1) $$\Delta i_{Norm}=\;\frac{i_{0}-i}{i_{0}}$$

**Figure 4:**
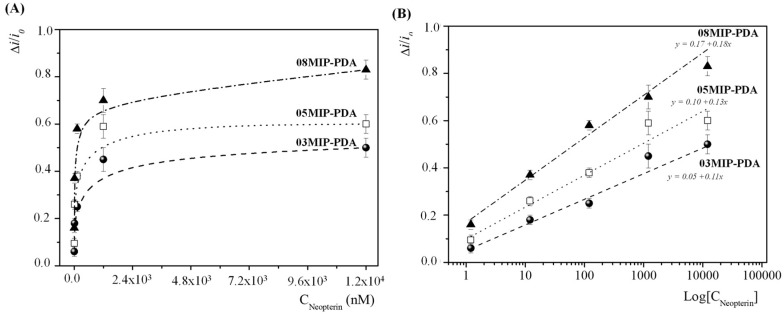
(**A**) Variation in the normalized peak current (Δi/i_0_) at various neopterin concentrations for the 3 different modified electrodes. (**B**) Semilogarithmic plot showing the linearized relationship between the normalized peak current (Δi/i_0_) versus the logarithm of different neopterin concentrations, for the 3 MIP-based electrodes.

Figure 4A shows the variation in the normalized current change (Δ*i_Norm_*) of the three prepared MIP-modified electrodes as a function of the neopterin concentration. In all cases, the trend is similar: at low neopterin concentrations (up to 120 nM), the response increases sharply, followed by a plateau at higher concentrations. As the neopterin concentration increases, the current of the redox probe progressively decreases due to the rebinding of neopterin within the imprinted cavities, which partially blocks the polymer layer and hinders the diffusion of the redox probe to the electrode surface. Consequently, the normalized signal variation increases sharply at low concentrations (up to ~120 nM) and then approaches a plateau at higher concentrations, indicating progressive saturation of the available binding sites. To better highlight the sensor behaviour over the full neopterin concentration range, the trend shown in Figure 4A has been replotted on a semi-logarithmic scale in Figure 4B, where the data exhibit a clear linear relationship with the logarithm of neopterin concentration.

Each MIP-modified electrode exhibited a linear response to neopterin in the range 1.2 nM–12 µM. The calibration equations were reported in the plots; R^2^ = 0.97, r = 0.99 are for all.

Such concentration-dependent behaviour is commonly observed in MIP sensors and is consistent with adsorption-based binding models, including Langmuir and related heterogeneous isotherms [42].

The detection limits (LODs), calculated as follows [43,44]:

(2) $$\mathrm{LOD}=\frac{3.3\;s}{slope}$$

with s = 0.02, were 0.60, 0.51, and 0.37 nM for 03MIP-PDA, 05MIP-PDA, and 08MIP-PDA, respectively.

The corresponding limits of quantification (LOQ), calculated according to the following definition:

(3) $$\mathrm{LOQ}=\frac{10\;s}{slope}$$

were 1.82, 1.54, and 1.11 nM, for 03MIP-PDA, 05MIP-PDA, and 08MIP-PDA, respectively.

All the calculated analytical parameters for the three MIP-modified electrodes are summarized in Table 2.

**Figure 5:**
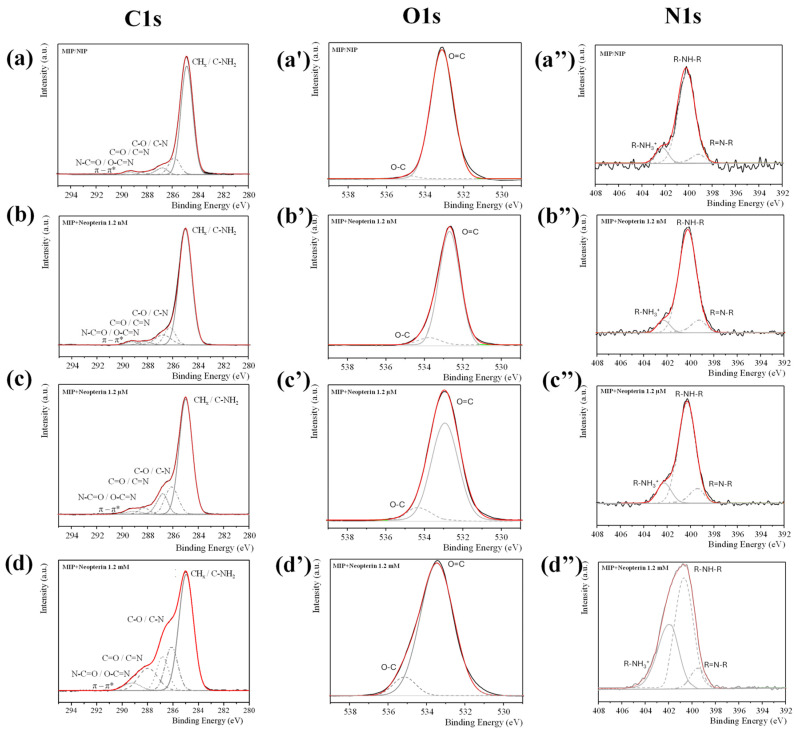
XPS spectra of C 1 s, O 1 s, and N 1 s for the 08MIP-PDA electrode: (**a**–**a″**) before exposure and for the NIP electrode; (**b**–**b″**) after exposure to 1.2 nM neopterin; (**c**–**c″**) after exposure to 1.2 μM neopterin; and (**d**–**d″**) after exposure to 1.2 mM neopterin.

Figure 5 shows the XPS spectra of the NIP and 08MIP-PDA electrodes before and after exposure to increasing neopterin concentrations (from 1.2 μM to 1.2 mM).

## 4. Discussion

The electrochemical results of the modified electrodes reflect the structural changes induced by template removal and neopterin rebinding. Indeed, this behaviour is consistent with the formation of three-dimensional (3D) imprinted cavities within the PDA layer, which act as channels allowing the redox probe to access the electrode surface. Consequently, probe diffusion is facilitated, resulting in an enhanced redox signal. As expected, the number of 3D cavities formed was directly related to the amount of neopterin used for MIP formation, leading to higher current responses for MIP electrodes prepared with greater neopterin concentrations.

The absence of redox peaks after electro-polymerization indicates complete coverage of the electrode surface by the PDA layer, which prevented probe diffusion. The partial restoration of the voltammetric signal after neopterin removal suggests the formation of cavities in the PDA layer that allow probe diffusion.

The decrease in sensor response upon incubation with increasing neopterin concentrations is consistent with the re-binding of neopterin molecules to the 3D cavities, which likely hinders probe diffusion and consequently affects the electron transfer process. The incomplete current recovery after acid washing is likely due to only partial restoration of the MIP cavities, as some template molecules remain hydrogen-bonded to the polymer matrix.

The comparison of the current variations indicates that the responses are larger for MIPs prepared with higher neopterin/DA ratios. The enhanced response of the MIP with higher neopterin content is attributed to a greater number of binding cavities available for interaction with the analyte. The plateau observed at higher neopterin concentrations arises from a decrease in the redox peak currents of the ferro/ferricyanide couple, as more imprinted sites are occupied.

In the semilogarithmic plot, the higher slope of 08MIP-PDA reflects its greater sensitivity, likely due to a larger number of binding cavities available for analyte interaction.

Finally, the CV results demonstrated that the ePDA-based MIP sensors exhibit a concentration-dependent response to neopterin. Overall, these findings highlight that tuning the template concentration may represent an effective strategy during MIP preparation to improve sensitivity and detection capability for neopterin. Among all prepared electrodes, the MIP-based sensor synthesized with the highest neopterin concentration (08MIP-PDA) showed the most optimized analytical performance.

To evaluate the chemistry at the MIP-modified electrode interface before and during the sensing interactions with the analyte molecule, XPS analysis was carried out exclusively on the best-performing MIP-modified electrode.

The evolution of XPS C 1 s components in the modified 08MIP-PDA electrode reveals an increase in C–N/C–O (286.0 eV) and C=O (288.0 eV) contributions with increasing neopterin concentration. At the same time, in Figure 5a′–d′, the component C=O at 531.3 eV of O 1 s XPS spectra increases upon neopterin rebinding, together with the C–O shifts towards higher binding energy (532.5–533.2 eV), which may be attributed to the formation of hydrogen bonding interactions between the MIP cavities and the neopterin molecules.

The overall O 1 s peak exhibits slight broadening and increased asymmetry, attributable to the overlap of PDA and neopterin signals. Furthermore, the XPS N 1 s spectra of PDA-based materials typically display components at about 399.7 eV, attributed to amine or imine groups, and at about 401.5 eV, corresponding to protonated amine or lactam species. Upon neopterin binding, an increase in the N 1 s contribution at about 401.5 eV may be associated with hydrogen bonding interactions and/or protonation of PDA nitrogen sites.

In conclusion, the proposed interpretation of the sensing mechanism is based on indirect electrochemical and spectroscopic evidence, and, therefore, it should be considered a plausible hypothesis rather than a definitive mechanistic demonstration.

## 5. Conclusions

A sensitive electrochemical sensor for neopterin detection was successfully developed using PDA-MIPs on a glassy carbon electrode. The PDA-MIP films were directly electropolymerized on the electrode surface by varying the ratio between dopamine and the neopterin template. CV, using ferricyanide as a redox probe, demonstrated that the MIPs effectively rebind neopterin over a broad concentration range (1.2 nM–1.2 mM), with a rapid response at low concentrations. The MIP containing the highest template content showed the best response, consistent with a greater number of specific binding sites.

XPS analysis further confirmed the chemical specificity of the MIP films. C 1 s, O 1 s, and N 1 s spectra revealed increases in C–N/C–O, C=O, and protonated nitrogen contributions upon neopterin rebinding, as well as shifts in binding energies consistent with hydrogen-bonding interactions. In addition, the overall O 1 s signal broadened and became asymmetric due to the superposition of PDA and neopterin contributions, further supporting successful template recognition.

These electrochemical and chemical results demonstrate the high sensitivity of the PDA-MIPs system, highlighting its potential as a promising device for the detection of neopterin. Although additional interference experiments were not conducted in the present study, the sensor is based on a molecularly imprinted polymer designed for selective recognition of neopterin, as previously reported in the literature [12,35]. Future studies will focus on further validating the selectivity of the proposed sensor by investigating the influence of structurally related metabolites (e.g., biopterin, creatinine, and uric acid) and other common components of biological fluids. In addition, recovery tests in a wider range of real samples, such as urine, serum, and cerebrospinal fluid, will be performed to further assess the practical applicability of the sensor. These investigations will provide additional evidence of specificity and support potential clinical or point-of-care applications.

## Figures and Tables

**Table 1:** Comparison of reported neopterin detection techniques, including recognition method, detected concentration range, limit of detection (LOD), main advantages, and limitations.

Detection Technique	Recognition Method	Concentration Range	LOD	Advantages	Limitations	Ref.
Potentiometric chemosensor	Electrochemically synthesized MIP	0.15–2.5 mM	22 µM	Simple electrochemical setup	Relatively high LOD	[12]
HPLC	Chromatographic separation	~10–1000 nM	~1–2 nM	Highly selective	Expensive, sample preparation	[17]
Radioimmunoassay (RIA)	Antibody-based recognition RIA	~50–2500 nML	~2–5 nM	Sensitive and validated	Radioactive reagents	[18]
Fluorescence Polarization Immunoassay (FPIA)	Antibody-based immunorecognition	~20–2000 m	~10 nM	Sensitive, relatively rapid, direct measurement	Requires fluorescence polarization instrumentation, with potential interference	[19]
Enzyme-Linked Immunosorbent Assay (ELISA)	Antibody-based immunorecognition	~2–100 nM	~2 nM	Easy to use	Multi-step	[20]
SERS-based sensor	Gold nanoparticle-based SERS substrate	0.1–10 µM	30 nM	Rapid, label-free, ultrasensitive	Specialized instrumentation, reproducibility challenges	[21]
Visible luminescence measurement (fluorescence spectroscopy)	Zn(II)–Eu(III) nanocluster	10 nM–10 µM	5 nM	Rapid, highly sensitive	Nanocluster synthesis, interference possible	[22]
SERS-based sensor	ZnO oxide films enhancing Raman signal; label-free molecular interaction	0.01–1 µM	5 nM	Ultrasensitive, label-free	Substrate reproducibility, specialized instrumentation	[25]
UV–vis spectrophotometry after solid-phase extraction	Surface-MIP on magnetic silica (MS)	3–300 nM	1.18 nM	Highly selective, reusable	Pre-extraction needed	[35]

**Table 2:** Calibration parameters, detection, and quantification limits for the three MIP-modified electrodes.

Modified- Electrode	Calibration Equation	Slope	Intercept	r	R^2^	LOD (nM)	LOQ (nM)
03MIP-PDA	*y* = 0.05 + 0.11x	0.11	0.05	0.99	0.97	0.60	1.82
05MIP-PDA	*y* = 0.10 + 0.13x	0.13	0.10	0.99	0.97	0.51	1.54
08MIP-PDA	y = 0.17 + 0.18x	0.18	0.17	0.99	0.97	0.37	1.11

## Data Availability

The original contributions presented in the study are included in the article, further inquiries can be directed to the corresponding author.
